# Preliminary Assessment of In Vivo Raman Spectroscopy Technique for Bone Quality Evaluation of Augmented Maxillary Sinus Floor

**DOI:** 10.3390/ijerph20064789

**Published:** 2023-03-08

**Authors:** Eduard Gatin, Pal Nagy, Stefan Marian Iordache, Ana-Maria Iordache, Catalin Romeo Luculescu, Valeriy Grygorovskyy

**Affiliations:** 1 Faculty of Medicine, University of Medicine and Pharmacy “Carol Davila”, Blv. Eroii Sanitari 8, Sector 5, 050474 Bucharest, Romania; 2 Faculty of Physics, DMSFAPA Department, University of Bucharest, 405 Atomistilor Str., 077125 Magurele, Romania; 3 Faculty of Dentistry, Periodontology Department, Semmelweiss University, 1085 Budapest, Hungary; 4 Optospintronics Department, National Institute for Research and Development for Optoelectronics—INOE 2000, 077125 Magurele, Romania; 5 CETAL Department, National Institute for Laser, Plasma and Radiation Physics, Atomistilor Str. 409, 077125 Magurele, Romania; 6 Research Institute for Traumatology and Orthopedics, 01601 Kyiv, Ukraine

**Keywords:** Raman spectroscopy, histomorphology, in vivo/ex vivo bone evaluation, early diagnosis, biospectroscopy, oral regenerative surgery

## Abstract

(1) Background: In oral surgery, bone regeneration is achieved through various types of bone grafts or bone substitutes and its success is usually analyzed by micro-computed tomography and histomorphometry. The aim of this study was to evaluate the usefulness of Raman spectroscopy as an alternative to other techniques for bone quality evaluation during a standard oral surgery procedure. (2) Methods: The preliminary evaluation of bone augmentation during maxillary sinus floor elevation oral surgery was performed by Raman spectroscopy for several (five) patients during and after the surgery and the results were compared with postoperative data from histomorphometry, EDX and SEM analysis. (3) Results: After analyzing all the results for the bone samples according to the four methods (Raman, EDX, SEM and Histology) that were used in our study, the obtained result of the investigation provided a good augmentation process for three of the patients and partly successful augmentation process for two of the patients. The primary evaluation using Raman spectroscopy (in vivo and ex vivo investigation) was confirmed by histological results, thus having a first step for validation of Raman as a new method of imaging for dentistry. (4) Conclusions: Our results show that Raman spectroscopy could provide fast and reliable insight on bone condition during augmentation of the maxillary sinus floor. We emphasize the advantages and drawbacks of the proposed techniques as its accuracy could increase by performing larger size clinical trials. Using the Raman mapping, the method can serve as an alternative to histology.

## 1. Introduction

Nowadays, a large number of surgical techniques have been used involving the implantation of various types of bone graft and/or bone substitutes in order to achieve bone regeneration. The majority of studies are evaluating the effects of different surgical procedures aimed at defecting filling with bone grafts and only employed clinical therapeutically measures, such as probing pocket depth, probing attachment level, radiological analysis and direct visualization, following surgical re-entry procedures. However, such approaches do not adequately evaluate bone regeneration, an outcome that requires systematic histologic investigation or more sensitive imaging methods [1,2,3].

In oral surgery or generally for an intraoperative pathologic diagnosis, the assessment of bone quality is traditionally performed by frozen section histopathology, which is labor intensive and time consuming. Indeed, a technique that streamlines the acquisition and evaluation of intraoperative histologic data may expedite surgical decision-making and shorten operative time. A new noninvasive and quick method to achieve information regarding chemical compounds from different tissues is highly desirable, and it will be the main challenge for the future developments in periodontal/oral regenerative surgery. Although timid, efforts are underway to transform this subjective art into a matter of objective and quantitative science.

The interest for the implementation of Raman spectroscopy in diagnosis and monitoring of diseases is very high because histopathology plays a central role in many diseases including solid cancers. In the field of general medicine, Stimulated Raman histology (SRH) was specially advanced as an emerging technology for investigating biological samples because it allows for more rapid acquisition and interpretation of intraoperative histopathologic data [4]. A new step forward was the introduction of Coherent Raman imaging (CRI), a label-free imaging modality with sub-cellular spatial resolution and molecule-specific contrast that possessed characteristics which could support the qualitative-to-quantitative transition of histopathology. These newly introduced methods are related to the modernization of histopathology, thus promoting the transformations that are underway [5].

The composition of bone can be described in terms of mineral phase and organic phase. Bone is a heterogeneous composite material consisting, in decreasing order, of a mineral phase composed from calcium phosphate (CP) compounds (OCP—octacalcium phosphate, HAP—hydroxyapatite, amorphous/crystalline phases), an organic phase (~90% type I collagen, ~5% non-collagenous proteins (NCPs), ~2% lipids by weight) and water. The relative proportions of these various components may vary with age, site, gender, disease and treatment [3,6]. All chemical compounds from bone composition are Raman sensitive.

We try to implement an investigation technique that is based on Raman spectroscopy for fast clinical investigation of bone condition in oral surgery. This nondestructive optical method based on laser beam scattering is able to characterize and differentiate initial normal cortical bone, initial augmentation material and final augmented bone. All bone components have distinct spectral features related to bone specimens’ concentration that are specific for each patient in close relation to medical status, periodontal disease stage and according to gingival phenotype [3].

Most studies involving Raman spectroscopy in dentistry and oral surgery reflect the interest that physicians have in assessment of dental enamel, periodontal ligaments or periodontal markers from saliva [7,8,9]. For those studies, investigation of samples is performed by using in vivo or ex vivo protocols. However, our study attempts to be a building block to identify future developments necessary to establish a close connection between emerging technology such as Raman spectroscopy and histology/histomorphometry and to bring it to the clinical end users. The goal of our study is to introduce a Raman spectroscopy based technique (noninvasive, quicker and accurate) as an alternative for bone evaluation. Using Raman mapping, the method can be promoted as an alternative to histology.

## 2. Materials and Methods

A group of five patients, under medical surveillance and with a very clear clinical status report for each one as healthy or periodontal problems, was selected for the present study. Evaluation for the patients was performed after the healing time period, approximately seven months from a maxillary sinus floor augmentation (MSFA) procedure. The difference between patients with healthy periodontium and those with previous periodontal disease is not relevant for the bone regeneration process in the sinus lift procedure. Some “differences” in bone properties are not distinguishable with the usual methods such as CBCT or X-ray, but they were evidenced with sensitive methods such as Raman spectroscopy [10]. Details regarding the group of patients are listed in Table 1.

**Figure 1:**
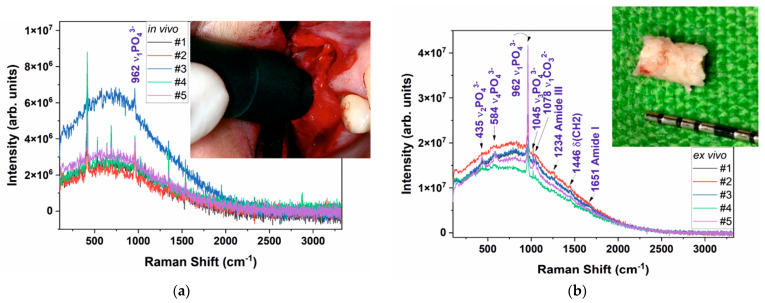
Raman spectra in 100–3300 cm^−1^ range acquired in vivo (**a**) and ex vivo (**b**) for all patients. The insets are showing the Raman probe head during in vivo acquisition (**a**) and bone sample collected from patient #3 for ex vivo investigation.

Subjects were selected among the patients referred for dental implant treatment at the region of the severely atrophic maxilla to the Department of Periodontology, Semmelweis University—Budapest. Due to the limited vertical bone height, all patients must undergo a maxillary sinus floor elevation (MSFA)—Figure 1a (inset). The patients needed to display at least one missing tooth at the region of the upper premolar or molar area, where the initial vertical bone height was <4 mm with a minimal horizontal width of 6 mm measured on preoperative CBCTs. If a patient presented more than one eligible site with a missing tooth, primarily the first molar tooth was involved and chosen as the site of interest. All patients signed an informed consent letter. A two-stage surgery protocol (MSFA with 7 months for the healing period, then a delayed implant placement during the re-entry procedure) was used. MSFA was performed under local anesthesia with full thickness flap preparation using the lateral window technique (Figure 1a (inset)) [11,12,13]. At this stage, an in vivo measurement was performed from the lateral bony wall of the sinus, which was later outlined as the bony window for the sinus entrance. This window was prepared with an ultrasonic device (Woodpecker, Guangxi, China) and gently mobilized inward and upward, creating a new horizontal ceiling (“trap-door” technique) for augmentation. Furthermore, a bony sample representing the original bone was harvested with the ultrasonic device. A saw shape head was used to take an about 2 × 3 mm cortical bony plate from the inferior border of the bony window. Then, inorganic bovine bone mineral (Cerabone^®^, Botiss Biomaterials GmbH, Zossen, Germany) with particle sizes ranging from 1.00 to 2.00 mm was hydrated with the subjects’ own blood and gently packed into the sinus. A resorbable membrane (Collprotect^®^, Botiss Biomaterials GmbH, Zossen, Germany) was used on the vestibular site to cover the bony window, and then the flap was sutured tension free.

A new mucoperiosteal flap was raised to expose the alveolar ridge at the second stage of the surgery in such a way as to reach the place of the former lateral bony window. At this stage, the second in vivo measurement was performed representing the healed (augmented) site, and then horizontal bony cylinders were harvested using a trephine (internal diameter 3 mm) (Meisinger, Neuss, Germany) under abundant saline irrigation. A biopsy was taken at 8 mm of depth penetrating directly into the newly formed bone without any contact with the old residual bone. At last, dental implants (ASTRA TECH Implant System™, Dentsply Implants, Skokie, IL, USA) were placed according to standard procedure from crestal insertion. All patients healed without any complication and were prosthetically rehabilitated.

To perform in vivo investigation for the patients, a “special head” was created for the Raman probe that can withstand steam autoclave sterilization according to standard protocol (Figure 1a). Prior to Raman measurements the selected area of the jawbone was prepared by blood aspiration, washed with standard saline solution and then dried with compressed air. During Raman scattering data collection for in vivo evaluation, we tried to avoid additional electromagnetic wave sources (no light), but even so, some luminescence contamination corresponding to krypton lamps was noticed. The Raman probe was handled in an orthogonal position on the jawbone surface chosen for examination (Figure 1a). For each patient, three Raman spectra were recorded [3,7,10].

In vivo examination was supplemented with bone biopsies for ex vivo evaluation. Both samples originating from the initial and the healed sites (size about 3 mm in diameter) were rinsed with standard saline and then stored in 70% ethyl alcohol solution. Prior to the ex vivo investigation, samples were rinsed with pure water and then a short air drying [3,10].

Raman spectroscopy investigation was performed by using a BTR111–785 RAMAN spectrometer device (λ = 785 nm, maximum output power P = 300 mW and spectral resolution as fine as 5 cm^−1^), in the Raman shift range 300–1800 cm^−1^ for both in vivo and ex vivo bone samples investigation. The integration time was 1000 ms and laser power was fitted for 10% from maximum output (30 mW). Raman probe was also in a vertical position for ex vivo evaluation, as was mentioned previously for in vivo investigation. Raman spectra were calibrated to the 520 cm^−1^ line of a Si (100) wafer, before and after data recording both in vivo and ex vivo measurements. Experimental data were recorded in the same geometrical conditions, for three points for in vivo measurements and also for three points during ex vivo bone sample evaluation in order to avoid local heating of the samples (recorded peak to peak according to the software) and no fluorescence contamination; data processing was obtained by using the equipment software and Bio–Rad KnowItAll 2017. Selected values for Raman peaks intensities were obtained after normalization (to the unit, average values) was applied to raw data (dark subtracted, not affected by noise, collected peak to peak). Differences in peak intensities on raw spectra reflect the differences in the quantities of the chemical components for investigated specimens. Sensitive qualitative/quantitative information may be obtained according to the Raman spectra shape (including the fluorescence information) using raw data (no flat line, without smoothing) [3,7].

Raman investigation highlights the peaks (Raman shift) for the main bone (cortical or cancellous type) components (chemical groups and elements) in order to evaluate the differences between bone tissue for the investigated patients (healthy, previous periodontitis or periodontitis). The following list (Table 2), in order of increasing wavenumber, shows the Raman bands (shifts) of bone tissue that are established to be relevant to our study and for future tracking and evaluation of the patients [3,10].

The next method of investigation for ex vivo evaluation of bone samples was EDS (Energy Dispersive X-ray spectroscopy) and followed by SEM (Scanning Electron Microscopy). The equipment employed to our study was a SEM microscope FEI Inspect S, equipped with a secondary electron detector in low vacuum and a solid state BSE detector, plus an auxiliary micro analytic EDS—Si(Li) radiation detector (EDAX Sapphire UTW, 130 eV resolution) [19,20].

The final method of evaluation involved in our study was histologic and upgraded with histomorphometry applied for bone samples harvested after the healing time period. The study was performed at the Research Institute for Traumatology and Orthopedics, Kyiv, Ukraine. Cerabone (granulated hydroxyapatite HAP) was used as augmentation material, in order to carry out the functions of volume replacement and serve as a matrix for apposition of a neogenic bone tissue. Quantitative aspects of bone tissue formation in implantation loci of various augmentation materials are very important. The purpose of our investigation was to determine histologic features, morphometric parameters and reparative osteogenesis efficiency in loci of implantation of a biomaterial “HAP-composite” in the preformed maxillary alveolar margin bone defects. Samples of alveolar margin from the maxillary were biopsied into small (the size was about 2 × 3 mm) fragments of a bone tissue formed on a place of the previous implantation of a biomaterial “HAP-composite”.

The removed fragments from the “HAP-composite” implantation locus were fixed in 70% alcohol solution, decalcinated in 5% nitric acid solution, permeated in alcohol solutions of an increasing concentration and imbedded in celloidin. Sections of 10 μm thick each were prepared from tissue blocks that were stained with hematoxylin, eosin, hematoxylin and picric acid as Van Gieson mixture. After evaluation of the qualitative histologic features of the tissues formed in the loci of the “HAP-composite” implantation, a morphometric investigation was carried out based on the planimetric method consisting in measuring separately areas constituted with the “HAP-composite” material and those with proper bone tissue.

## 3. Results and Discussions

**Figure 2:**
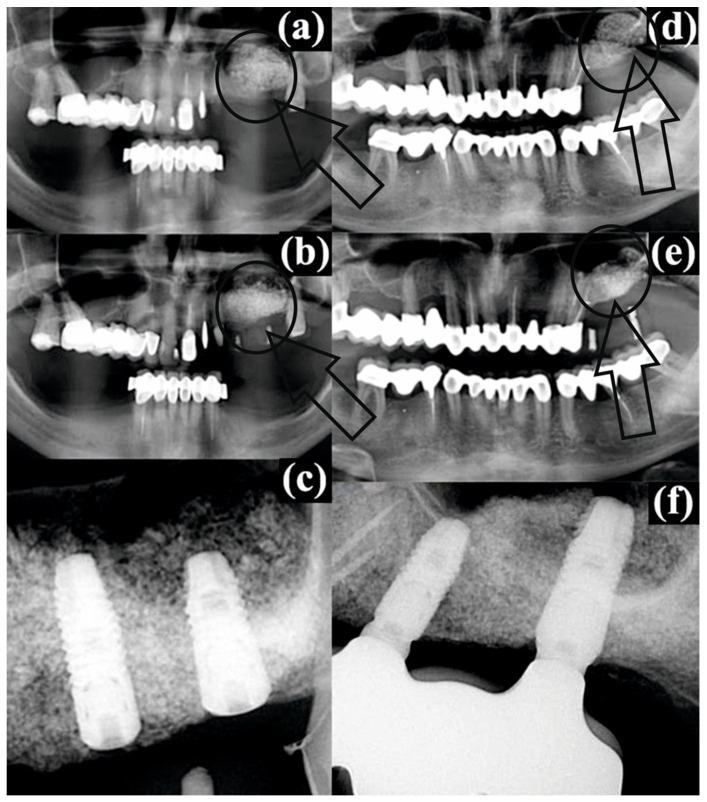
Radiographic images of patient #2: (**a**) the augmented bone in the left sinus immediately after sinus floor augmentation-highlighted with arrow and circle, (**b**) postoperative conditions after 4 months of healing-highlighted with arrow and circle, (**c**) dental implants in the augmented bone at 7 months after augmentation; and patient #3: (**d**) the augmented bone in the left sinus immediately after sinus floor augmentation-highlighted with arrow and circle, (**e**) postoperative conditions after 4 months of healing-highlighted with arrow and circle, (**f**) dental implants in the augmented bone at 7 months after augmentation. The augmentation loci are encircled and arrow are pointing to the regions of interest.

As was mentioned in the previous section, for all patients as a standard procedure a CBCT evaluation was performed (before surgery, post operatory and after the healing period/with dental implants). The results are presented in Figure 2.

For patient #2, the augmented bone material in the left sinus immediately after sinus floor augmentation (Figure 2a) can be clearly observed as a spongy aerated filling compared with the compact structure from the rest of periodontium. After four months of healing, the conformation was the same, but with a small advance of the filling to the left side and sticking to the sinus floor wall (Figure 2b, pointed by a small arrow) with the same gray shades very similar to the image of a lattice. Regarding patient #3, the same conformation can be noticed after the sinus floor augmentation (Figure 2d) as for patient #2. However, after the healing period, for the radiographic image (Figure 2e) a “white spot” can be observed as a compact area of bone substitute (pointed by a small arrow) with no lattice structure.

According to the Raman spectra depicted in Figure 1 for the in vivo and ex vivo measurements, the results are presented in a condensed form and detailed in Table 3.

Considering that for bone tissue (both old tissue as well as new bone tissue) collagen (Col) is representative and that the Raman peak of HAP at 962 cm^−1^ (HAP _962_) is representative for the augmentation material noted prior to the start of our discussion, it is useful to introduce as a parameter the ratio R = I _Col_/I _962_ (corresponding intensities for Raman peaks, according to Table 2).

An important observation we need to address is that all in vivo Raman spectra are affected by krypton (Kr) lamp light. The intensity of the Kr line at 415 cm^−1^ varies from one patient to another according to the lab conditions. However, for all in vivo spectra, the ν_1_ PO_4_^3−^ Raman mode is present in all spectra. Both in vivo and ex vivo spectra are affected by a broad luminescence peak that is centered at about 700 cm^−1^ [21,22]. The luminescence peak is related to collagen, most probably to the second order of collagen I emission maxima [23,24,25,26,27].

For all measurements, the ex vivo spectra are stronger, and more Raman modes are visible for PO_4_^3−^, CO_3_^2−^ and collagen. Considering that for bone tissue (both old tissue as well as new bone tissue) collagen (Col) is representative and that the Raman peak of HAP at 962 cm^−1^ (HAP_962_) is representative for the augmentation material and bone prior to our discussion section, it is useful to introduce R = I_Col_/I_962_ as a parameter (corresponding intensities for Raman peaks, according to Table 3). As a general remark, the rates (R) respect the same trend for the in vivo respective as for the ex vivo evaluation for all patients (only with smaller values for in vivo experiments) as a result of the different light collection geometry. Regarding the value behavior, some “rules” regarding the status of the bone augmentation process can be observed:

(a) For a balanced bone augmentation process, R is focused on the mild range area values (0.50 ÷ 0.70, as Q_3_ from total scale). We suggest that this is the status for a good augmentation process that is corresponding to patients #1, #2 and #4.
(b) For a nonbalanced bone augmentation process, R is placed on extreme values (0.30 ÷ 0.40 or 0.80 ÷ 0.99, as Q_1_ or Q_4_ from total scale). We emphasize that this is the status for a questionable (partly successful) augmentation process, corresponding to patients #3 and #5.

To conclude the discussion for the Raman evaluation, we must take in consideration values from the columns I _Col_ and I _Pyr_ associated with Table 3. Information derived from those values is connected and regards the augmentation process. According to the status of the bone augmentation process (new bone tissue), the following behavior for I_Col_ and I_Pyr_ can be observed:

(i) Medium values in both columns (40 ÷ 65 a. u.) associated with high values (I_Col_, about 70 a. u.) and with small (I_Pyr,_ about 20 a. u.) values are noticed for patients #1, #2 and #4;
(ii) High values in both columns or small values in both columns are noticed clearly for patients #3 and #5.

The analysis of Raman spectroscopy data shows a successful bone augmentation procedure for patients #1, #2 and #4. On the other hand, for patients #3 and #5, we appreciate that the success is questionable or partial successful.

An important parameter regarding bone quality is the fraction Ca/P in terms of weight (W) or atoms (A). The results obtained for the bone samples after augmentation (healing time) using the EDX method are depicted in Table 4.

A good balance between immature bone (amorphous phase HAP) and mature bone (crystalline phase HAP) is around 1.33 ÷ 1.40 [3]. An increased value for this fraction is normal for an augmentation bone process because of the bone substitute (higher crystalline phase). In case of very high values, as noticed for patients #3 and #5, we emphasized that bone substitute remained focused in some areas, not spread in the collage matrix or existing bone tissue.

**Figure 3:**
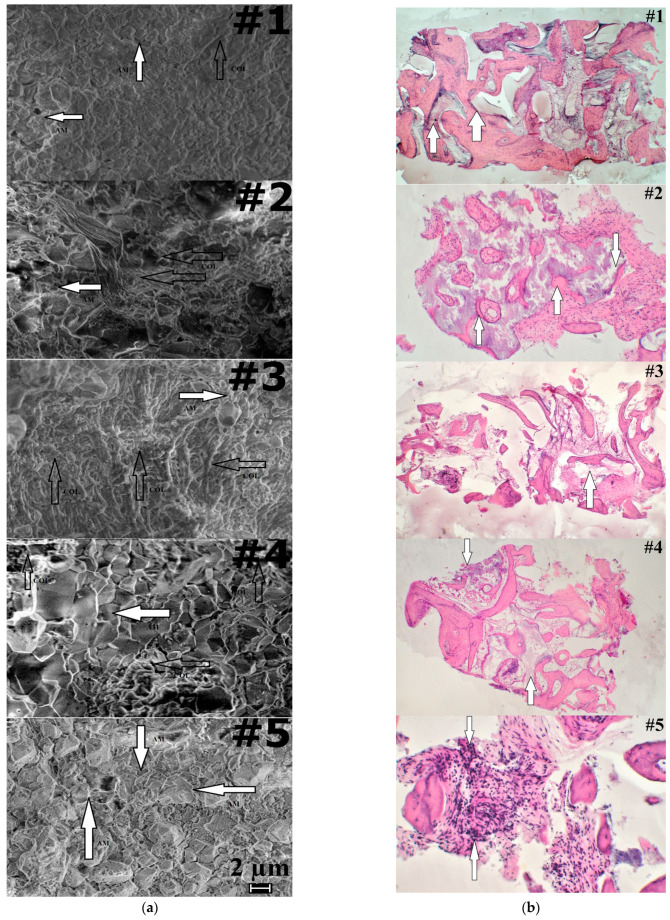
(**a**) SEM micrographs of bone samples, magnification 10,000X. Legend: COL—collagen (white arrows), AM—bone augmentation material (black arrows); (**b**) Histology, morphological changes reflecting the condition of the biological augmentation process using HAP (Cerabone) as bone substitute. Changes are indicated by arrows for the specific areas.

A good proof regarding our evaluation of the bone augmentation process is offered by the SEM micrographs presented in Figure 3a. For patients #1, #2 and #4, it can be clearly noticed that there is an alternation between areas of collagen (COL) and augmentation material (AM), thus for those patients we conclude that the bone augmentation process was successful. Regarding patients #3 and #5, we have the opposite situation. Large compact areas of COL or AM, with no penetrations or links between them, can be observed, which is a mark for a not totally successful bone augmentation process. The SEM investigation is in good agreement with our previous conclusions provided by Raman and EDX analysis.

The final proof to sustain and support the results from Raman expertise is histology twofold with a histomorphometry investigation. Results from the histology investigation are presented in Figure 3b for each of the five patients. In the Figure 3a, patient #1, at a magnification of 30X, the aggregation of implant materials HAP—composite (pointed by arrows) among excrescences of a neogenic bone tissue, offers a general view of the tissue sample in the place of the HAP-composite implantation. Signs of an ongoing phase of bone resorption in the form of an overlay of osteoclast-like multinucleated cells are more common on the surfaces of implant granules are also observed.

In the case of the healthy patient in Figure 3b, for patient #2, at 75X magnification, the granules of implant material of HAP—composite are surrounded by excrescences of a fibrous tissue (pointed by arrows). The granules of the augmentation material are in the majority of places in contact with a fibrous tissue. For the fibrous tissue located in the spaces between the beams, there were varying sizes that had different density (often insignificant) accumulations of mononuclear cells and macrophages. Deep ingrowth of connective tissue vessels into a massif of the HAP—composite implant material with a formation of the minimum quantities of an osteoid-bony tissue (pointed by arrows) on the inner surfaces are also noticeable.

For the third patient, as shown in Figure 3b, a minor (small) quantity of the HAP-composite implant material formed polymorphic accumulations of bizarre shape (pointed by arrow) that remained among the fibrous and bone tissue. In addition, when appearing at 30X magnification, the implant material formed polymorphic accumulations, often having a bizarre shape.

In Figure 3b, for patient #4 we notice that large HAP—composite implant granules (pointed by arrows) are surrounded by bone and fibrous tissue (the last prevails). Among the fibrous tissue located in the spaces between the beams, there were varying sizes, different density (often insignificant) accumulations of mononuclear cells and macrophages. Moreover, 30X magnification was used to obtain a general view of the tissue sample around the “HAP-composite” implantation site.

As for the fifth patient in Figure 3a, small focal mononuclear inflammatory infiltrates (pointed by arrows) among the fibrous tissue suggest in-growing into the depth of the HAP—composite material area. It can be observed as a productive nonspecific inflammation of low activity (magnification 150X). Table 5 summarizes in a quantitative manner the evaluation of the morphological changes noticed in Figure 3b.

The last column in Table 5 reflects the area ratio (R_H_) of “bone tissue/implanted material” as it was defined. For patients #1, #2 and #4, a balance for *R_H_* as medium values (1.2222 ÷ 2.5082) can be observed, but for patients #3 and #5, the trend is for extreme values (smaller or higher, 0.6970 and 4.7692).

Values presented in Table 5 for R_H_ represent ***the confirmation (above all)*** for the evaluation and confirm results/conclusions from the Raman expertise. Comparing the obtained results with the radiographic images from the CBCT for patients #2 and #3, we conclude that those methods are more accurate in bone quality evaluation. We emphasize that for patients #1, #2, #4 it was a successful augmentation, and for patients #3 and #5 it was just “partial”. Using just the primary evaluation with the CBCT, all patients were considered recovered and healed from the medical point of view.

Moreover, as we aimed for a complete evaluation, we consider the average values and indicators of variation in the coefficient “Ratio R*_H_*” bone tissue—implant material in the compared groups with different “gingival phenotype”, which offer the possibility to establish differences in the average values, and the average parameter of the coefficient in the group of patients with a “thin” gingival phenotype (X ± sx = 2.229 ± 2.215 with n = 3) exceeded that in the group of persons with a “thick” gingival phenotype (X ± sx = 2.148 ±0.509 with n = 2); however, rather high parameters of variation and a small number of observations do not allow us to consider the difference in the average values as reliable between the two categories (since we only have five patients).

**Figure 4:**
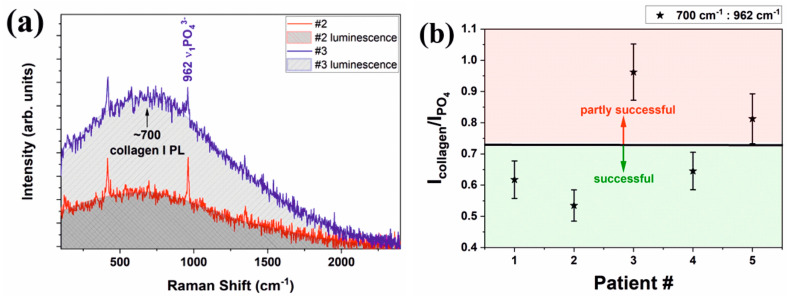
(**a**) The in vivo Raman spectra of patients #2 and #3 that have different status showing the bands used in quantification; (**b**) the results of quantifications performed in this study based solely on in vivo Raman spectra.

All ex vivo spectra have a better signal-to-noise ratio and most common Raman bands for both organic and mineral are easily discernible. For in vivo measurements, all Raman spectra are noisy and only the strongest band at about 962 cm^−1^ related to the ν_1_PO_4_^3−^ vibration is clearly visible along with the broad luminescence centered at about 700 cm^−1^ that we assigned to the second order luminescence from collagen type I [26,27] (Figure 4a). Patient #2 and patient #3 are belonging to different “classes”; #3 is a patient with certain previous periodontal disease, while #2 is a certain healthy patient. All others are former previous periodontal.

In the past, we tried to differentiate ex vivo between periodontitis affected and healthy periodontium by Raman spectroscopy. We tried to compare the ratio of different Raman bands, and we performed different data smoothing and normalization that could provide useful information [3]. However, for the in vivo measurement, our former analysis could not apply due to severalissues: noisy spectra, bone contamination with body fluids, stray light contamination (clinics’ lights, krypton lamps in our case).

The ratio of the second order luminescence band at ~700 cm^−1^ to the strongest phosphate band at about 962 cm^−1^ that is related to ν_1_PO_4_^3−^ vibration will provide a ratio between the amount of collagen and crystalline apatite. This ratio is not expected to be linear as luminescence and Raman scattering are probing different energy levels but, nevertheless, it is sensitive to the bone health condition. The results for the ratio of collagen to apatite obtained by in vivo Raman spectroscopy are plotted in the Figure 4b for all patients. An estimated error of 20% is expected for our data. The ratios under 0.7–0.725 are characteristic for healthy patients, while a ratio that exceeds that value is representative for periodontal patients.

The strength of this study is the fact that we obtained a fast and clear separation by Raman spectroscopy between normal bone, augmented bone and sectors having augmentation problems during a standard oral surgery. Those results were confirmed by the SEM and histologic investigations. The limitation of our study is the fact that our work samples were few, just five patients. Increasing the number of patients could lead to a better comparison between the two categories (we could extract more data and maybe perform a Raman mapping on the samples), and this can be the subject for a future study.

## 4. Conclusions

After putting side by side all the results for the bone samples evaluation according to the four methods (Raman, SEM, EDAX and Histology) that were used in our study, the results of investigation were cross-confirmed.

Raman spectroscopy used as a spearhead method in our study offered good results for bone sample evaluation for both in vivo and ex vivo investigation. Results obtained were supported by the rest of the methods, all leading to the same evaluation results for the patients. The advantage of the Raman method is that the requested time is substantially smaller and no special sample conditions for preparation is needed compared to histology. For in vivo use, it is much less invasive than the classic methods (CBCT, X rays) and higher in accuracy. Regarding accuracy of the method, we calculated it to be fair enough as considering the small number of patients and limited comparison points. The aim of the study was just to introduce Raman spectroscopy as an alternative to histology. By increasing the number of patients and creating a larger database storage (with multivariate statistical analysis), the Raman spectroscopy can be improved and turned into an emergent tool as an alternative to histology for bone quality evaluation in periodontal investigation and oral surgery. For the moment, references regarding this topic are very limited.

## Figures and Tables

**Table 1:** List of patients involved in the study with clinical remarks; specifications regarding bone samples: (• healthy), (• previous periodontal).

Patient Number	Gender	Age (Years)	Healthy	Periodontal	Gingival Phenotype	Bone Type
#1	M	58	---	Previously	thick	more cortical
#2	F	56	Yes	---	thin	more cortical
#3	M	64	---	Previously	thin	more cortical
#4	F	50	---	Previously	thick	more cortical
#5	M	70	---	Previously	thin	more cortical

**Table 2:** Targeted Raman shift for bone specimens.

Raman Shift	Characteristics	Assignment	References
430–450 cm^−1^	very strong	ν_2_ PO_4_^3−^, shoulder	[14,15]
955–960 cm^−1^ 955 cm^−1^ 957 cm^−1^	very strong	Extensive mineral immature bone; ν_1_ PO_4_^3−^, P–O phase; ν_1_ PO_4_^3−^, extensive HPO_4_^2−^	[15]
960–965 cm^−1^ 963 cm^−1^	very strong	Mineral mature bone; ν_1_PO_4_^3−^ tetrahedral internal mode.	[15]
1023 cm^−1^	Strong	PPi (P_2_O_7_^4−^), inorganic pyrophosphate; symmetric P-O stretch modes of PO_3_^2−^ moieties; ν_5_ PO_3_ and of P–O–P bridging.	[16,17,18]
1070 cm^−1^ 1076 cm^−1^	Strong	Mineral bone B—type carbonate HAP CO_3_^2−^ (ν_1_) overlap PO_4_^3−^ (ν_3_) overlap	[14]

**Table 3:** Summarized Raman results. Ratio (R) of normalized Raman peaks intensities, peaks intensities (I) according to Raman shift. Summarized results: normalized, average values (three points for in vivo */ex vivo *, data acquisition), standard deviation SD included. Legend: col—collagen, 962—crystalline HAP, Pyr—pyrophosphate, Color coding: (• healthy), (• previous periodontal), (• augmentation problem). The results are presented as measured values; average value; SD (standard deviation).

Patient	Status (Ex Vivo/In Vivo)	R = I_Col_/I_962_	I_Col_	I_Pyr_
#1	ex vivo *	0.67, 0.51, 0.63; 0.60 SD = 0.08	33.98, 37.13, 66.25; 45.78 SD = 17.79	34.45, 63.07, 48.91; 48.81 SD = 14.31
	vivo *	0.67, 0.61, 0.58; 0.62 SD = 0.04	43.48, 49.90, 56.20; 49.86 SD = 6.36	23.34, 43.34, 56.45; 41.04 SD = 16.67
#2	ex vivo	0.72, 0.70, 0.65; 0.69 SD = 0.03	78.47, 82.28, 14.53; 58.42 SD = 38.06	15.93, 69.27, 19.40; 34.86 SD = 29.84
	vivo	0.60, 0.65, 0.53; 0.65 SD = 0.05	60.70, 72.47, 62.30; 65.15 SD = 6.38	60.39, 48.20, 58.34; 55.64 SD = 6.52
#3	ex vivo	0.81, 0.78, 0.85; 0.81 SD = 0.03	73.93, 79.30, 83.83; 79.02 SD = 4.95	76.64, 81.84, 78.83; 79.10 SD = 2.61
	vivo	0.96, 1.03, 0.99; 0.99 SD = 0.09	83.49, 88.46,79.18; 83.71 SD = 4.64	73.97, 69.67, 81.23; 74.95 SD = 5.84
#4	ex vivo	0.68, 0.73, 0.70; 0.70 SD = 0.02	68.15, 72.66, 74.14; 71.65 SD = 3.12	67.75, 70.41, 69.53; 69.23 SD = 1.35
	vivo	0.68, 0.64, 0.70; 0.67 SD = 0.03	38.88, 32.93, 46.23; 39.34 SD = 6.66	37.51, 48.65, 51.62; 45.92 SD = 7.43
#5	ex vivo	0.38, 0.42, 0.40; 0.40 SD = 0.02	38.60, 41.91, 40.81; 40.44 SD = 1.68	39.27, 43.72, 41.92; 41.63 SD = 2.23
	vivo	0.81, 0.87, 0.91; 0.86 SD = 0.08	58.34, 79.18, 62.20; 66.57 SD = 11.08	50.65, 69.02, 68.87; 62.84 SD = 10.56

**Table 4:** Ca/P fraction; summarized results according EDX method (three measurements for each sample, standard deviation STD included; in green/blue are the ‘successful’ patients and in red the ones that have augmentation problem).

Patient Number	Ca/P Ratio W/A	Mean Value	STD
#1	1.92, 2.09, 1.99 1.62, 1.59, 1.51	1.96 1.57	0.13 0.05
#2	1.56, 1.79, 2.06 1.20, 1.38, 1.59	1.80 1.39	0.25 0.19
#3	1.73, 2.45, 2.23 1.33, 1.89, 1.72	2.13 1.64	0.36 0.28
#4	1.89, 1.95, 1.91 1.20, 1.38, 1.59	1.91 1.39	0.03 0.19
#5	1.98, 2.17, 2.23 1.53, 1.68, 1.81	2.12 1.67	0.13 0.14

**Table 5:** Morphometric data of the patients for the examined hydroxyapatite (HAP-composite) specimens that were implanted. (in green/ blue are the ‘succesfull’ patients and in red the ones that have augmentation problems).

Morphometric Data			
Patient	Summarized Area of Implanted Material Sections, mm^2^ *	Summarized Area of Bone Tissue Sections, mm^2^ *	“Bone Tissue—Implanted Material” Areas Ratio (R*_H_*)
1.	302 squares 4 × 4 mm * 1.1446	540 squares 4 × 4 mm * 2.0466	1.7881
2.	36 0.1364	44 0.1668	1.2222
3 .	39 0.1478	186 0.7049	4.7692
4.	122 0.4624	306 1,1597	2.5082
5.	165 0.6254	115 0.4359	0.6970

* Note: Summarized areas of implanted augmentation material and bone tissue were estimated on the microphoto of specimen histologic preparation separately by square grid with sides 4 × 4 mm, which was calibrated by an object-micrometer of 1000 mm long. Calculated area of square 4 × 4 mm was equal to 0.00379 mm^2^.

## Data Availability

Not applicable.
